# The generation and gender shifts in medicine: an exploratory survey of internal medicine physicians

**DOI:** 10.1186/1472-6963-6-55

**Published:** 2006-05-05

**Authors:** Emily Jovic, Jean E Wallace, Jane Lemaire

**Affiliations:** 1 Department of Sociology, University of Western Ontario, London, Ontario, N6A 5C2, Canada; 2 Department of Sociology, University of Calgary, 2500 University Drive NW, Calgary, Alberta, T2N 1N4, Canada; 3 Department of Medicine, University of Calgary, Health Sciences Centre, 3330 Hospital Drive NW, Calgary, Alberta, T2N 4N1, Canada

## Abstract

**Background:**

Two striking demographic shifts evident in today's workforce are also apparent in the medical profession. One is the entry of a new generation of physicians, Gen Xers, and the other is the influx of women. Both shifts are argued to have significant implications for recruitment and retention because of assumptions regarding the younger generation's and women's attitudes towards work and patient care. This paper explores two questions regarding the generations: (1) How do Baby Boomer and Generation X physicians perceive the generation shift in work attitudes and behaviours? and (2) Do Baby Boomer and Generation X physicians differ significantly in their work hours and work attitudes regarding patient care and life balance? Gen Xers include those born between 1965 and 1980; Baby Boomers are those born between 1945 and 1964. We also ask: Do female and male Generation X physicians differ significantly in their work hours and work attitudes regarding patient care and life balance?

**Methods:**

We conducted exploratory interviews with 54 physicians and residents from the Department of Medicine (response rate 91%) and asked about their perceptions regarding the generation and gender shifts in medicine. We limit the analyses to interview responses of 34 Baby Boomers and 18 Generation Xers. We also sent questionnaires to Department members (response rate 66%), and this analysis is limited to 87 Baby Boomers' and 65 Generation Xers' responses.

**Results:**

The qualitative interview data suggest significant generation and gender shifts in physicians' attitudes. Baby Boomers generally view Gen Xer physicians as less committed to their medical careers. The quantitative questionnaire data suggest that there are few significant differences in the generations' and genders' reports of work-life balance, work hours and attitudes towards patient care.

**Conclusion:**

A combined qualitative and quantitative approach to the generation shift and gender shift in medicine is helpful in revealing that the widely held assumptions are not necessarily reflective of any significant differences in actual work attitudes or behaviours of Boomer and Gen X physicians or of the younger generation of women entering medicine.

## Background

Two striking demographic shifts, evident in today's workforce in general, are also apparent in the medical profession. One is the entry of a new generation of physicians, Generation Xers, and the other is the influx of women into the medical profession. Gen Xers include the twenty- and thirty-something women and men who were born between 1965 and 1980. In contrast, the Baby Boomer generation refers to the large cohort born between 1945 and 1964. Growing attention is being paid to generation and gender shifts in the work orientations of practicing physicians. Titles such as "Are You Ready for Generation X?" [1], "Bridging the Generation Gap(s)" [2], and "Managing Gen Xers Strategically" [3] illustrate concerns being raised in the literature with regard to the latest cohort of young professionals. Similarly, titles such as "When Most Doctors are Women: What Lies Ahead?"[4] and "A Force to Contend With: The Gender Gap Closes in Medical Schools" [5] reflect the growing attention to the entry of more women into medicine.

Why is it important to examine generation and gender shifts in physicians' attitudes and experiences? First, as Shields and Shields [6] note in their paper on working with Generation X physicians, most of the assumptions about generation and gender shifts are supported at best by anecdotal evidence rather than empirical data. We need more research to understand and substantiate existing conceptions about the generations and genders. Douglas Coupland popularized the term "Generation X" in his 1991 novel, where he characterized the generation as cynical, naïve, and disillusioned with the world and materialism, respecting no one and valuing nothing [7]. In review articles, we see a number of commonly identified characteristics of Generation Xers that include: a desire for autonomy and flexible schedules; preference for the latest technology; emphasis on close friends or family more than material success; emphasis on personal growth, expressing creativity and developing new and portable skills; insecurity and cynicism about organizations; and more open attitudes towards diversity [1,3,6]. In contrast, Baby Boomers are described as holding an exceptionally strong work ethic, characterized by hard work, long hours and loyalty to their employers [8,9]. Gen Xers criticize Boomers as overly cautious, competitive, blindly loyal and hierarchy worshipping [10]. Boomers criticize the younger generation for their lack of involvement in their jobs, lack of commitment to their careers and overall lack of work ethic. They describe Gen Xers as a generation who "couldn't care less" [10]. It has been suggested that the most rigid generational differences are related to the role that work plays in one's life [11]. Whereas it seems members of the Baby Boomer generation define themselves as doctors first, the younger physicians see practicing medicine as only one part of their identity, and perhaps not even the most important part. The Baby Boomer generation is totally committed to medicine – it is who and what they are, whereas the Gen Xers might define themselves as perhaps a physician, gardener, marathon runner and Cub Scout leader. This fundamental difference in how the generations define themselves results in Gen Xers placing greater emphasis on lifestyle choices and making sure they have more time for family and leisure.

Second, it is also important to examine generation and gender shifts in medicine because physician shortages in many medical specialties are driving recruitment and retention [1,6,12]. As the Boomer generation ages and retires, Generation Xers will be expected to fill these positions, despite a relative deficiency in manpower, compared with the preceding generation. The literature suggests that both Gen Xers and women are more concerned about work-life balance and quality of life than the Boomers in general. This is partly evidenced by women working and preferring to work fewer hours per than men, and an increasing number of younger men also preferring to work less than the traditional 50 to 60 hours a week in order to spend more time with their family [13]. As Washburn notes, "'Work your butt off and make a lot of money' may not attract applicants as it once did. Family time and balance between work and play may mean more to Gen-X physicians than large incomes" [1]. As another author suggests: "Younger physicians know (the current economic slump notwithstanding) there's a coming shortage of doctors and they're not about to be exploited – work longer hours, be loyal as dogs – as the boomers were" [11].

Third, it is important to examine these shifts because increasing numbers of women are entering medicine, and Canada is no exception in this regard. For example, in 1981 women represented 13% of Canadian practicing physicians [14], and by 2005, they represented 31.3% [15]. These numbers are comparable to those reported for the United States [16] and several European countries, such as the Netherlands [17], the United Kingdom [18] and the Nordic countries [19]. Researchers have referred to the growing representation of female doctors as the "feminization of medicine" [4], and are asking how this demographic trend will affect patient care, health care systems and physician careers. Some research suggests that female doctors are more likely than their male counterparts to engage patients as active partners in their care and to meet the challenges of the trend towards multidisciplinary team approaches to patient care [4]. Research also shows that women and men tend to enter into different areas of specialization and types of practice [4]. For decades, sociologists have examined the gendered segregation of occupations, and they suggest that gender differences in occupations, specialties and work settings may be linked to differences in work and family values that lead women to select jobs and fields that best facilitate work-family balance [19,20]. Much of this research frames the question of why men and women end up in different jobs as hinging on two competing theoretical frameworks that reflect gendered choices or constraints. Basically, the choice or supply-side explanation suggests that gender differences in careers are due to the choices of individual women. That is, women tend to choose different occupations, specialties and work settings than men because women hold different work and family values and interests that lead them to select jobs and fields that best facilitate work-family balance [20]. The constraint or demand-side explanation focuses on the choices and behaviors of employers rather than employees. These explanations emphasize how institutional barriers or discrimination make it difficult for workers, particularly women, to balance work and family responsibilities, which may then lead to gender inequalities at work. For example in medicine, different working conditions, career structures and career orientations and different opportunities for combining a medical career and family life are associated with various medical specialties [21,22]. Based on the results of her study, Gjerberg [23] suggests that both individual choice and the constraints associated with different specialties are responsible for variations in the work-family interface for physicians, which is also consistent with the broader literature in this area [24].

In general, women's entry into medicine has had a major influence on issues of work-life balance and quality of work life: "Women in medicine have forged new pathways to allow physicians to balance career and family responsibilities" [4]. Compared to men, women work fewer hours per week, see fewer patients (and provide fewer services), take time off to have children, are likely to leave the profession sooner, and are less inclined to join professional organizations [5,13]. Women in medicine will continue to pressure policy makers to foster flexibility and enable balance between professional and personal life, helping both women and men in medicine to meet responsibilities to their children, their parents and themselves. As a result, the gender shift will influence health care systems, as maternity leaves, leaves of absence to raise small children or care for aging parents, and part-time practices significantly reduce physicians' work hours and affect where and how they practice medicine.

## Methods

### Aim

In light of the issues raised above, the aim of this paper is to answer three questions: (1) How do Baby Boomer and Generation X physicians perceive the generation shift in physicians' work attitudes and behaviours? Finding that they indicate a shift in perception, we then ask (2) Do Baby Boomer and Generation X physicians differ significantly in their work hours and work attitudes regarding patient care and life balance? and (3) Do female and male Generation X physicians differ significantly in their work hours and work attitudes regarding patient care and life balance?

### Setting

Data for this study were collected from doctors and residents from a university and health region based Department of Medicine in a large, metropolitan city in Western Canada. Most are involved primarily in clinical work and many are also part of the teaching and research programs sponsored by the university. Staff physicians usually function as independent professionals with their work patterns cast by the patient-care centered, academic and/or scholarly responsibilities and expectations of their division. In contrast, residents have little control over their work environment as their educational curriculum is based on national objectives of training, which must be completed by the end of the training period. A large percentage of a resident's work time consists of caring directly for patients in a hospital or outpatient setting and includes after hours on call work. In this particular Department of Medicine, 23.6% of the physicians are women, and in Canada, women represent 25.2% of internal medicine physicians [15].

### Design

Two data collection strategies were used in this study: (1) semi-structured interviews with a quota sample of 54 physicians and residents; and (2) a short, structured mail-out questionnaire sent to all 275 physicians and residents in the department. The qualitative interview data are used to answer the first research question posed in this paper. The questionnaire data are used to answer Question 2 and Question 3.

### Semi-structured interviews (qualitative analysis)

#### Sample

We invited 59 physicians and residents to participate in the interviews and 54 agreed to take part (response rate 91%). Those who declined stated concerns over lack of time (n = 2), confidentiality (n = 2) or inability to represent department views because of type of practice (n = 1). A quota sampling strategy was used to select potential participants based on gender, division, rank, site/hospital affiliation, scholarly activity and family status. For this paper, we limit the analysis to the responses of 34 Baby Boomer and 18 Generation X physicians and residents who participated in the interviews. The two participants excluded from the analysis do not fall into either generation being examined in this study. Table 1 provides a demographic profile of the Baby Boomer and Generation X doctors who participated in the interviews.

#### Data collection

Interviews were conducted at the participant's convenience, usually during work hours and at their place of employment. The interview questions were mostly open-ended and exploratory in nature, allowing respondents to describe their work experiences and attitudes in their own words. Interviews generally lasted approximately one hour although they ranged in length from 30 to 95 minutes. The interviewer typed participants' responses in a word processing program on a notebook computer during the interview and reviewed her notes immediately after to correct any typographical errors or omissions. The particularly relevant questions for this paper are: (1) "As the demographics of the medical profession changes, what effects do you think the younger generation of physicians will have on the profession and/or the way things currently work in your department? Do you think this change in a positive or negative direction?" and (2) "What about the increasing number of women in medicine? How do you think that will impact the profession and your department? Positive or negative?"

#### Data analysis

We used *HyperResearch*, a software package for qualitative data analysis, to analyze the interview responses. This software enables researchers to code and retrieve data and cases for analysis. The authors of this article independently reviewed the responses to the questions posed to participants about their attitudes towards the influx of the younger generation of physicians and women into the medical profession. We did not use pre-established categories for analyzing the interview data. Rather, we used an inductive strategy through open and selective coding to derive the predominant themes reflected in the interview transcripts. We then went back to the original data and coded each response so as to compute frequencies for the number of participants who mentioned each theme.

### Questionnaires (quantitative analyses)

#### Sample

In December 2004, 275 questionnaires were sent to all physicians and residents in the Department of Medicine and 182 surveys were returned yielding a 66% response rate. For this paper, we limit the quantitative analysis to 87 Baby Boomer and 65 Gen X physicians. The remaining 30 respondents are excluded from this analysis because they were born prior to 1945. Table 2 presents demographic information for the Baby Boomer and Generation X doctors who completed the questionnaire.

#### Data collection

Most items in the questionnaire were closed-ended, Likert items from established measures in the field. We coded the responses such that "Strongly Agree" and "Agree" were coded 1 and all other responses were coded 0. This allows us to present the findings as the percentage of respondents agreeing with a particular response. This coding scheme generates more easily interpretable results than reporting a value between 1 ("Strongly Disagree") and 5 ("Strongly Agree").

Work hours were measured by a single question that asked, "On average, in a typical week, how many hours do you work in total (including evenings and weekends)?" Respondents estimated how many hours a week they worked at the office and how many hours a week at home. Given the nature of their jobs, "at the office" may include a variety of locations, such as a hospital, clinic or the university. We computed the total average number of hours they work per week by summing their estimates of their weekly work hours at the office and at home. Patient care was measured using two Likert items: "I really care what happens to my patients" and "I feel I am positively influencing other peoples' lives through my work" [25]. Respondents indicated the extent to which they agreed with the statements and their responses were coded 1 if they agreed or strongly agreed and 0 otherwise. These items are used as proxies for elements of physicians' commitment to medicine. They are consistent with items used in classic measures of affective work commitment and involvement (e.g., [26,27]) with regard to caring about or being involved with aspects of one's work.

Work-life balance was measured using four different Likert items: "I feel I have a pretty balanced life" [28]; "The demands of my work interfere with my home and family life" [29]; "My family responsibilities interfere with my work" [29], and; "I feel I have enough time to do the things I want to do" [30]. Respondents indicated the extent to which they agreed with the statements, which were coded 1 if they agreed or strongly agreed and 0 otherwise.

#### Data analysis

We analyzed the quantitative data using SPSS 13.0. We computed t-tests to assess the statistical significance of differences in the attitudes and work experiences of Boomers versus Gen Xers and female versus male Gen Xers. Because of the relatively small sample size for some of the comparisons, statistically significant results at the .10 level (one-tailed tests) are presented (see Cohen [31]).

## Results

### How do Baby Boomer and Generation X physicians perceive the generation shift in physicians' work attitudes and behaviors?

In the analysis of the interviews, three key themes emerged: work-life balance, work ethic (hours) and commitment to medicine. The percentages in brackets represent how many participants mentioned this particular theme and participants often mentioned more than one theme.

The predominant theme raised repeatedly by both Baby Boomer (64%) and Generation X doctors (67%) is the greater emphasis placed on work-family balance and lifestyle by both female and male Gen Xer physicians. Both Boomers (53%) and Gen Xers (61%) suggest the push for more life balance is linked to the growing numbers of women entering medical school and their desire for flexibility and a more manageable workload because of greater family responsibilities. Upon completing increasingly lengthy medical training, many women are at a crux in terms of contemplating the time to have a family. A female Gen Xer physician with an infant explains: "*When we come out of training, it takes so long, there's only a small window to have our babies*." Thus, in the early years of their medical careers, some women may work less than full time to accommodate their young children's needs. Nonetheless, many participants acknowledge that the current trend does not only involve women. For example, a female Boomer suggests: "*It's the men that are equally as interested in having a life and knowing their family and participation in their family life, even if it means that their work has to take up less of their time. It's a delightful change*." In contrast, the older generation of doctors is viewed as extremely committed to and involved in their careers, often at the expense of their family and personal lives. A male Boomer doctor felt there were changes in the profession resulting from more emphasis being placed on life balance: "*We're no longer these egotistical, godlike, you know, revered people. Now we're human beings*".

Both groups of doctors recognize a generational divide in perceptions of attitudes toward the practice of medicine and the work ethic. Half of the Boomers (50%) and two-thirds (67%) of the Gen Xers mentioned how the younger generation tends to work fewer hours and is perceived as not working as hard as previous generations. Generation Xers suggest that Baby Boomer doctors place their career front and center in their identities and they hold a work ethic to match: "*I think the older generation, if I can say that, medicine was who they were... So there was a very different mentality as far as work was concerned. They were going to work 8 to 8 every day, be on call as much as they had to*." One Gen Xer describes this as an artifact of the Boomers' personal circumstances as follows: "*the model of the male physician and the stay-at-home wife who looked after kids and ran the household, and said physician was able to work really long hours, 7 days a week, and provided a lot of continuity of care*".

Conversely, while Gen Xer physicians feel their medical careers are important, they do not necessarily place it at the forefront as the only aspect of who they are. As a Gen Xer explains: "*You don't have to be defined by the job you do. Being a parent is good; being able to balance is good. ... I'm not working 365 days a year. I don't need to do that to be a better doctor*." The long work hours, expected as simply part of medical practice by previous generations' doctors, no longer holds in the view of the younger generation. A Gen Xer puts it simply: "*Everyone in general is just not willing to devote 100 percent of their life to medicine anymore*."

The emphasis Generation Xers place on achieving a more balanced life is often interpreted by Boomers (64%) as an indicator of their lack of commitment and unwillingness to work. Boomers generally believe that the work ethic has eroded and there has been a significant decline in the value and importance of work, as indicated by the attitudes and behaviors of the Gen Xers.

Nearly one third of the Gen Xers (28%) mention that they believe they are viewed as less committed to their careers or that their careers are less important because they strive for a balanced life. A Boomer commented: "*What I'm seeing is a lot of the upcoming fellows and students don't work as hard as my cohort did in order to get the career they want. There may be benefits to that ... but it appears they are not working as hard*." This seeming disinterest in working "hard" (i.e., long hours) translates into a perceived lack of commitment to work and to medicine, as this Boomer explains: "*At the training level, you're a bit frustrated because you get the impression that they're not as devoted. I'm not sure 'devoted' is the right word, committed ... to learning medicine*." However, a Gen Xer defends their position, stating: "*I think they *[the younger generation] *are just as committed, but believe more in the importance of being well-rounded and having an outside life*." Gen Xer doctors suggest that Baby Boomers work, or have worked, too many hours, sometimes at the expense of their families and personal lives, and that sort of lifestyle is simply not for them. Thus, from the Baby Boomer perspective, working long hours is tied to commitment to medicine, perhaps reflecting the centrality of "being a doctor" to their self-perception, whereas for Gen Xers, these links do not appear to be as strong.

### Do Baby Boomer and Generation X physicians differ significantly in their work hours and work attitudes regarding patient care and life balance?

Given the qualitative interview results suggest a notable generation shift in the work attitudes of Baby Boomer and Generation X physicians, we compared their self-reports of work hours, patient care and work-life balance. First, regarding work hours, Table 3 shows that Boomers (mean = 51.2 hours) report working nearly 10 hours *less *each week at work than Gen Xers (mean = 60.7 hours). Boomers report working about an hour and a half more at home each week than Gen Xers. However, overall, Gen Xers actually work nearly an additional full work day (7.95 hours) each week. This significant difference may be capturing different career stages, rather than generational differences. The Gen X group includes residents, who work notoriously long hours (though supposedly *fewer *hours than Boomers did at that stage), as well as physicians just establishing their practices. In order to explore whether the younger residents are inflating Generation Xer hours, we reran the analysis without them. When residents are excluded from the analysis, both generations work an average of 61 hours a week.

The results in Table 3 also show that there are no significant differences between Boomers' and Gen Xers' attitudes towards patient care. Both groups of physicians have similarly strong, positive orientations toward patient care. Lastly, Boomers and Gen Xers generally report similar amounts of balance, or lack thereof. Less than half of the respondents (42% for Boomers and 49% for Gen Xers) feel they have balanced lives. Moreover, nearly two-thirds (65%) of both groups feel that their work interferes with their home and family life. Only 20% of respondents report they have enough time to do the things they want to do. Nearly one-third (29%) of the Boomer respondents report family responsibilities interfere with their work, whereas only 12% of the Gen Xers feel that way.

### Do female and male Generation X physicians differ significantly in their work hours and work attitudes regarding patient care and life balance?

Table 3 also contains results for the same nine items from the generational analysis for physicians' work hours, patient care and work-life balance with comparisons by gender. Overall, there are very few significant gender differences.

The results in Table 3 show that female Gen Xers (mean = 70.25) report working slightly more hours a week than male Gen Xers (mean = 67.81), although this difference is not statistically significant. There also appears to be no significant gender differences in how Gen X physicians approach patient care on two fronts, caring for patients and feeling they are positively influencing other peoples' lives. Lastly, the results suggest that women and men report similar amounts of balance. It should be noted, however, that only about half feel they have balanced lives, and about two-thirds (67% of the women and 63% of the men) feel their work interferes with family. A much greater proportion of the women (21%) than men (3%) feel that their family responsibilities interfere with work. This is a statistically significant difference. When we only examine the results for parents, we see that 67% of the nine mothers, compared to *none *of the 17 fathers, report family-to-work conflict. Finally, most of the respondents report they simply do not have enough time to do all of the things they want to do. Again, more women (88%) than men (72%) feel this way, which is in line with the literature [32,33].

## Discussion

The tenor of the literature and the ways of thinking reported in the workplace suggest that there are growing concerns about the assumed dissimilarities and potential tensions between the generations and the genders. We set out to explore whether the generations themselves perceive such a shift in their work attitudes and whether they report significant differences in their actual work attitudes and experiences. From the interview data, we find that Baby Boomer physicians, consistent with the literature, believe that the younger generation of doctors is more concerned about having a balanced life and a lifestyle, is less committed to medicine and does not work as hard as previous generations. In contrast, Generation X physicians feel they are just as committed to their medical careers as previous generations. They do not believe, however, that working long hours is a necessarily valid indicator of their work ethic or commitment. Other aspects of their lives are also important in how they define themselves and spend their time.

Through the Gen Xers' emphasis and attempts to achieve a balanced life, Boomers believe the younger generation is less committed to their careers. Boomers seem to view the relationship between the work and family domains as zero-sum, or independent, where greater commitment to one means less commitment to the other. This does not appear to be the case for Gen Xers who instead see both domains as interdependent, converging and important in contributing to their overall well-being and sense of life balance [34]. As a result, Gen Xers feel they can be committed to both simultaneously, whereas Boomers experience greater role conflict between work and family.

Despite perceptions of a generational shift, the quantitative analysis suggests that Baby Boomers and Generation Xers report similar attitudes and experiences with respect to patient care and work-life balance. And, when residents are excluded from the analysis, both generations average 61 work hours a week. These findings suggest that, regardless of differences in perceptions about the two generations, they are more similar in certain ways than they realize. These similarities may reflect the extensive professional socialization that physicians receive throughout their medical training that promotes the internalization of the same core work values and behaviors associated with practicing medicine, regardless of which generation they belong to. Throughout this training, a common understanding of occupational knowledge and norms are formed that likely lead to a convergence of values and practice styles amongst physicians [35-37]. These findings have important implications for "bridging the generation gap." Through knowledge translation of the results of studies such as this one, it is possible to educate the generations about their similar work habits and attitudes.

The influx of women into medicine has brought issues such as work-life balance to the forefront, since women must often juggle their medical careers alongside family responsibilities. In addition, the literature suggests potential differences between female and male physicians' orientations towards their patients, such that women may show more empathy and concern for the well-being of their patients than men [4,16]. We therefore compared the work attitudes and experiences of women and men from Generation X. From this analysis, it appears that female and male Gen X physicians do not differ significantly in their work hours or orientations toward patient care, contrary to some of the anecdotal assumptions in the literature and the workplace [4].

The literature clearly shows that female physicians are far more likely than male physicians to work part-time [5,18,36], which makes it even more surprising that their work hours should be as similar as they are. For example, further analysis of the data in this study show that 15% of the women worked less than 40 hours a week at the office compared to 6% of the men. In other studies, reports as high as 50% of women practice medicine part time (less than 40 hours a week), depending on their specialization and work setting, compared to less than 10% of men [18,36,38]. McMurray et al. [36] report, however, that despite the significant difference in the *number *of hours worked, they found no gender differences in the *proportions *of time women and men spend in patient-related or other work-related activities, such as administration or teaching. More importantly, perhaps, are findings that suggest that part-time, primary-care physicians are more productive compared to their full-time counterparts and provide at least the same quality of patient care or better, with similar reports of patient satisfaction [38,39]. The literature suggests there is a growing decline in full-time equivalents of physician services, especially in areas where women are entering in greater numbers (e.g., family practice, obstetrics and gynaecology) and a sustained decline in the number of applicants where women do not apply (e.g., surgery) [5]. While the trend toward part-time clinical practice may not harm patient outcomes, it is occurring in the midst of a growing physician shortage in North America.

In professional work, work hours are often equated with commitment and worth, where longer hours mean a more dedicated professional [17,23] who provides better quality and continuity of care [13]. The lack of part-time options in combination with the expectation of long work hours calls for a fundamental change in medical culture so that doctors, women and men alike, can be more involved with their families without it being detrimental to their careers. Heiligers and Hingstman [13] found that 50% of all specialists in their study preferred a part-time working arrangement and similar results have been reported elsewhere (e.g., [40,41]). They conclude that a large proportion of physicians feel a need for a reduction in work hours and this need is not restricted to female doctors. An obvious consequence of reducing work hours would necessitate increasing the intake of medical students and a greater degree of flexibility in employment settings in order to optimize efficiency in the use of their human resources.

It is also interesting to note that the survey data indicate neither women nor men of Generation X are experiencing large amounts of balance in their lives, despite Baby Boomer perceptions to the contrary. Moreover, similar proportions of Gen X women and Baby Boomers report that their family responsibilities interfere with their work. Further analysis (results not shown) shows that approximately one-third of both women and men Boomers, and one quarter of Gen X women report family-to-work conflict, whereas virtually none of the Gen X men do. One interpretation of these results may be that Boomer parents are more likely to perceive and perhaps even resent non-work responsibilities interfering with their careers and career success. In contrast, Gen Xers may welcome the integration of their work and family lives and may be less likely to perceive the interaction between the two as a negative "interference". Further research should examine the extent to which the two generations have positive or negative attitudes towards the work-family interface and the interaction between their two life domains.

The results also suggest that young female physicians are experiencing slightly greater challenges in terms of balancing their lives, particularly with respect to family responsibilities interfering with work and having the time to do the things they want. There are several reasons why becoming a mother can make combining a balanced life with a satisfying career very difficult for professional women. One is that women professionals generally, and women doctors specifically, are more likely to have a spouse with a similarly demanding professional career, whereas male professionals and doctors are more likely to have spouses with less demanding jobs [23,42]. This results in greater work-family conflict for professional women because they still retain a majority of the responsibility for household and family demands, even when they work comparable hours as their husbands [23,40,42]. For example, a recent study shows that male physicians perform only 19% of childcare duties and 26% of household duties whereas female physicians perform two-thirds of both [43]. These findings are consistent with those reported elsewhere [44,45]. In addition, women are still expected to put family before career, while society does not place the same expectations on men [17]. On the contrary, men in professional careers are expected to place their priority on their careers as they fulfil the "male breadwinner role" and their wives, regardless of whether they are professionals or not, primarily care for the household and family.

Women in medicine make different adjustments to manage the challenges of combining family life and a medical career [19,23]. Some mothers strive to "have it all" or "satisfice" [46] by attempting to achieve success in both their careers and family, without choosing one over the other. This often results in feelings of role overload, conflict and a general sense of imbalance as mothers attempt to satisfy two competing sets of demands and responsibilities. Other mothers "scale back" [47] their careers by significantly reducing their work hours and responsibilities. As indicated above, women are more likely to work part time than men and the primary reason is in order to better balance work and family. As Budig and England [48] suggest, however, following the theory of compensating differentials, mothers may trade off certain job rewards, such as higher wages or career advancements, for jobs that make it easier to combine work and family. Thus, work-life balance for women often has economic and career costs that may take the form of forgone wages and delayed career advancement [49]. Those who work full time and sacrifice their family life are generally viewed as committed to their careers, and those who work part time and give priority to their family are seen to be less committed [21]. Whereas men and members of the earlier generations tended to do the former, women and members of the younger generations tend to do the latter, thereby confounding the shift in generational attitudes with gender and contributing to the attitudinal rift between the generations [50,51].

## Conclusion

In this paper, we empirically investigated two recent generations of physicians' and both genders' attitudes towards patient care and work life balance, and this is an important contribution. By combining qualitative and quantitative data to study the generation shift and gender shift in medicine, we demonstrated that typical, widely held assumptions are not necessarily accurate or reflective of any significant differences in the actual work attitudes or behaviours of physicians from these groups. Future research should examine a broader range of work attitudes and experiences (e.g., career commitment, sense of job security, attitudes and use of technology). As well, it would be interesting to explore whether similar patterns observed between the current generations are repeated as the new Generation Y begins to enter the workforce in larger numbers.

## Competing interests

The author(s) declare that they have no competing interests.

## Authors' contributions

EJ, JW and JL contributed equally to the writing of this paper.

JL and JW jointly conceived the idea for the study and drafted the measurement instruments.

EJ collected and analysed the interview data and did the quantitative analysis.

JW collected the quantitative data and was responsible for data entry.

## Pre-publication history

The pre-publication history for this paper can be accessed here:



## Figures and Tables

**Table 1:** Characteristics of interview participants

	Baby Boomers (N = 34)	Generation X (N = 18)
Gender	Male n = 18 (53%) Female n = 16 (47%)	Male n = 8 (44%) Female n = 10 (56%)
Age	Mean = 48.15 (SD = 4.58)	Mean = 35.06 (SD = 3.69)
Years in department	Mean = 11.12 (SD = 7.00)	Mean = 3.79 (SD = 2.04)
Marital status	Lives alone n = 4 (12%) Lives with partner n = 30 (88%)	Lives alone n = 3 (17%) Lives with partner n = 15 (83%)
Parental status	Has no children n = 5 (15%) Has children n = 29 (85%)	Has no children n = 12 (67%) Has children n = 6 (33%)
% Residents	Resident n = 2 (6%)	Resident n = 4 (22%)

**Table 2:** Characteristics of questionnaire participants

	Baby Boomers (N = 87)	Generation X (N = 65)
Gender	Male n = 64 (74%) Female n = 23 (26%)	Male n = 32 (49%) Female n = 33 (51%)
Age	Mean = 49.3 (SD = 5.5)	Mean = 34.5 (SD = 3.5) Missing n = 1
Years in department	Mean = 12.4 (SD = 8.4)	Mean = 3.5 (SD = 2.3) Missing n = 2
Marital status	Lives alone n = 8 (9%) Lives with partner n = 78 (91%) Missing n = 1	Lives alone n = 9 (14%) Lives with partner n = 55 (86%) Missing n = 1
Parental status	Has no children n = 14 (16%) Has children n = 72 (84%) Missing n = 1	Has no children n = 38 (60%) Has children n = 25 (40%) Missing n = 2
% Residents	Resident n = 3 (3%)	Resident n = 24 (38%)

**Table 3:** Mean difference tests between Baby Boomers vs. Generation Xers and Gen X women vs. Gen X men

Survey Items	Boomers (n = 87)	Gen Xers (n = 65)	Gen X women (n = 33)	Gen X men (n = 32)
Work hours (including residents)				
Hours per week at office	51.2	60.7*	61.3	60.2
Hours per week at home	9.9	8.3*	9.0	7.6
Hours per week total	61.1	69.1*	70.3	67.8
				
Work hours (excluding residents)^a^				
Hours per week at office	50.9	53.8	51.9	55.3
Hours per week at home	9.9	7.6	8.3	7.1
Hours per week total	60.8	61.4	60.2	62.4†
				
Patient care				
I really care what happens to my patients.	88%	94%	94%	94%
I feel I am positively influencing other people's lives through my work.	91%	92%	91%	94%
				
Life balance				
I feel I have a pretty balanced life.	42%	49%	48%	50%
The demands of my work interfere with my home and family life.	65%	65%	67%	63%
My family responsibilities interfere with my work.	29%	12%*	21%	3%†
I feel I have enough time to do the things I want to.	19%	20%	12%	28%†

* indicates a statistically significant difference at the .10 level between Baby Boomer and Generation X physicians

† indicates a statistically significant difference at the .10 level between Generation X women and Generation X men

^a ^The following are excluded from these results: 3 Boomer residents; 24 Gen X residents; 14 Gen X female residents and 10 Gen X male residents
